# Renal clearance of fluorescent agents can compromise image‐guided surgery along the urinary tract

**DOI:** 10.1111/bju.16804

**Published:** 2025-06-30

**Authors:** Anne‐Claire Berrens, Tessa Buckle, Matthias N. van Oosterom, Leon J. Slof, Belle M. Melsert, Jakko A. Nieuwenhuijzen, Esther M.K. Wit, Pim J. van Leeuwen, Henk G. van der Poel, Fijs W.B. van Leeuwen

**Affiliations:** ^1^ Department of Urology Netherlands Cancer Institute – Antoni van Leeuwenhoek Hospital Amsterdam The Netherlands; ^2^ Department of Urology Amsterdam University Medical Center, Location VUmc Amsterdam The Netherlands; ^3^ Interventional Molecular Imaging Laboratory Leiden University Medical Center Leiden The Netherlands

**Keywords:** image‐guided surgery, fluorescence guided surgery, radioguided surgery, surgical margin assessment, prostate cancer, PSMA, PSMA‐targeted surgery, fluorescein

## Abstract

**Objectives:**

To study the effect of renally cleared fluorescent agents on image‐guided surgery along the urinary tract by using the renally cleared, non‐tumour‐specific, fluorescent dye fluorescein.

**Subjects and Methods:**

Sixteen patients who underwent robot‐assisted radical prostatectomy (RARP) with lymph node dissection received an intradermal injection of fluorescein. The slow‐release of the fluorescein from the skin into the lymph‐ and bloodstream were used as a pharmacokinetic model for slow release from receptor‐targeted agents. The presence of fluorescein in the urine and the surgical dissection planes around the prostate (representative of cancer margins) during RARP were evaluated. Suction, gauze and irrigation were used to try and reduce fluorescent background signals according to standard operating protocol.

**Results:**

Fluorescein was detected in the urine in the bedside catheter bag after a median of 1.3 h after agent administration and in the surgical field after opening the bladder neck as part of RARP (median of 2.4 h after injection). Suction and application of gauze helped to reduce contamination, but suction combined with irrigation with lukewarm NaCl 0.9% was shown to be most effective. Fluorescein accumulation was seen in the tissue surrounding the bladder neck in 80% of patients.

**Conclusions:**

Renally excreted fluorescent agents risk contamination of the surgical field and possible dissection margins along the urinary tract, a feature that, without proper counter measures, could compromise the accuracy of intra‐operative imaging by creating false‐positive findings. A clear example of this was the observed bladder neck staining with fluorescein.

**Abbreviations**
ICG: indocyanine green
IQR: interquartile range
PSMA: prostate‐specific membrane antigen
RARP: robot‐assisted radical prostatectomy

## Introduction

**Fig. 1:**
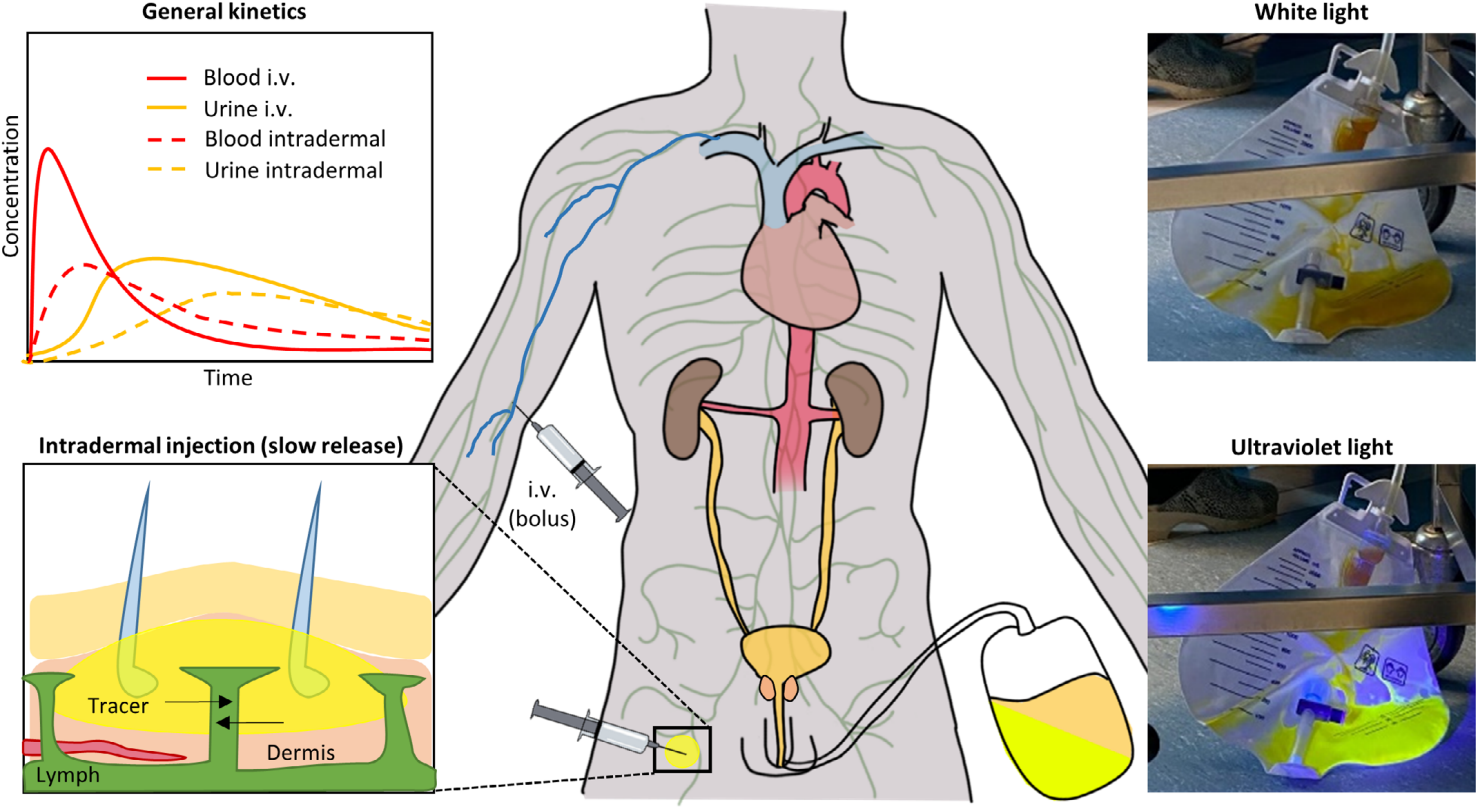
General pharmacokinetics based on ADME Encyclopedia and Buckle et al. [10, 33]. Intravenous injection facilitates immediate presence of agents in the (early peak). Intradermal injection creates a more stationary agent deposition at the site of injection, followed by slow release from the tissue. As a result, the latter can be used to mimic receptor‐targeted agents, such as PSMA‐targeted agents, that remain available over a prolonged period of time. As fluorescein is renally cleared, it was visible in the urine in the bedside catheter bag using ultraviolet flashlight.

The use of intra‐operative molecular imaging represents a significant evolution in surgical procedures. Various fluorescent agents are used for the identification/visualisation of different anatomical or molecular features in different indications. For example, i.v. applied agents can be used for angiography (indocyanine green [ICG] or fluorescein), visualisation of bile efflux, and disturbances therein (ICG, mebrofenin and visualisation of ureters (fluorescein) [1, 2, 3]. Prostate‐specific membrane antigen (PSMA) targeting has proven to be a popular receptor‐targeted approach in primary and salvage prostate cancer surgery [4]. Imaging labels (chelates and/or fluorescent dyes) used to functionalise targeting vectors such as PSMA can substantially impact the pharmacokinetic clearance profile [5]. After the body is perfused with a bolus of agent‐containing blood through i.v. injection (Fig. 1), tumour tissue and tumour‐specific receptors can be targeted [6]. During this same first‐pass perfusion, some of the agent accumulates in healthy background tissue. This effect is dependent on serum binding [7]] and dosing [8, 9]. The initial targeting phase is then followed by slow release of non‐receptor‐bound agent residing in background tissues [10]. This clearance of background tissue may take place over a prolonged period, in some cases requiring a time interval of 17 days between agent injection and surgery [11].

Renal excretion is perhaps the most important and common route of excretion for imaging agents, such as ^68^Ga‐PSMA‐11, ^99m^Tc‐PSMA I&S [4], 111In‐PSMA I&T [12], IS‐002 [8], OTL78 [9] and IR800‐IAB2M [11]. A drawback of the use of agents cleared by the kidneys is that contaminated urine can stain the surgical field [13]. When the signal is assumed to be related to the cancerous tissue, non‐tumour‐specific uptake is likely to compromise surgical margin assessment along the urinary tract and promote overtreatment. Indeed, various studies have shown that the use of ^68^Ga‐PSMA‐11, ^99m^Tc‐PSMA I&S, IS‐002, OTL78 and IR800‐IAB2M during PSMA‐targeted prostate cancer surgery poses a risk of false‐positive results due to contaminated urine [8, 9, 11, 14, 15]. These same studies indicate that this effect becomes more prominent with higher dosing. Importantly, therapeutic dosing (mg/kg) tends to promote contamination when compared to the micro‐dosing commonly used for radio agents (≤100 μg/patient) [8, 9, 11, 14].

Given the rising interest in PSMA‐targeted prostate cancer surgery, it is imperative to increase our general understanding of the potential false‐positive effects caused by urine contamination [4]. To gain insight into the effect of the presence of imaging agent in urine during surgery, we used intradermal injections of the fluorescent dye fluorescein to mimic and visualise the clearance of non‐bound PSMA agent (Fig. 1) [10, 16, 17].

## Methods

A total of 16 patients (Table 1) were included as part of a single‐arm, single‐centre, prospective, feasibility study (NCT05120973) [18]. The study was approved by the local ethics committee and all patients provided written informed consent. The patients underwent robot‐assisted radical prostatectomy (RARP) with extended pelvic lymph node dissection and a sentinel node procedure between March 2022 and August 2023.

**Table 1:** Patient demographics.

Characteristic	*n* = 16
Median (IQR) age at surgery, years	66.5 (61.0–69.0)
Median (IQR) initial PSA, ng/mL	15.3 (7.8–21.9)
Clinical T stage, *n* (%)	
T1	3 (19)
T2	11 (69)
T2b	1 (6)
T3a	1 (6)
Radiological T stage, *n* (%)	
T2	5 (31)
T2b	1 (6)
T3a	7 (44)
T3b	3 (19)
Radiological N stage PSMA PET/CT, *n* (%)	
N0	15 (94)
N1	1 (6)
Biopsy ISUP grade, *n* (%)	
2	4 (25)
3	7 (44)
4	1 (6)
5	4 (25)
Median (IQR) risk of LNI according to Briganti 2012 [19], %	18.9 (12.5–33.7)
Median (IQR) time injection fluorescein to first intraoperative imaging, h	2.4 (2.0–2.6)
Median (IQR) time injection fluorescein to urine visible in bedside catheter bag, h	1.3 (1.2–1.7)
Pathological T stage, *n* (%)	
T2	5 (31)
T2c	6 (38)
T3a	1 (6)
T3b	4 (25)
Pathological N stage, *n* (%)	
N0	10 (62)
N1	6 (38)
Pathological ISUP grade	
2	5 (31)
3	9 (56)
4	1 (6)
5	1 (6)
Surgical margin status, *n* (%)	
R0	11 (69)
R1	5 (31)

IQR, interquartile range; ISUP, International Society of Urological Pathology; LNI, lymph node involvement; *n*, number.

### Fluorescein Administration

Fluorescein (80 mg [~1 mg/kg]; 4 mL 2%) was injected unilaterally into the dermis in two deposits of 2 mL each. The injection was performed in the operating room, directly after general anaesthesia. The injection was unilaterally placed, either at the medial and lateral side of the upper leg (*n* = 8) or at the left or right lower quadrant of the abdominal wall (*n* = 8). As part of a separate objective of the prospective study protocol, intraprostatic ^99m^Tc‐ICG‐nanocolloid was administered [18]. Because of the hepatobiliary clearance pattern of ICG, this was not included in this assessment of urine contamination.

### Fluorescein Mechanism of Action and Pharmacokinetics

Fluorescein is excited by blue light (λ_max ex_ = 488 nm) and emits light that appears yellowish‐green (λ_max em_ = 515 nm) [20]. Fluorescein can enter the bloodstream either after a systemic bolus injection (routine angiographic use) or following slow release from tissue deposits over time. The latter deposits can either be the result of non‐specific accumulations in the interstitial space (0.5 L/kg) that follow bolus injection or can be the direct result of intradermal fluorescein deposition, as was the case in this prospective study protocol. Following intradermal injection of fluorescein, it took approximately 10 min to visualise the (micro)‐lymphatics of the skin [20, 21]. Fluorescein undergoes rapid metabolism to fluorescein monoglucuronide (80% within 1 h and almost all within 4–5 h [20]) and is mainly eliminated via renal excretion. Glomerular filtration provides a key pathway for excretion of the 10%–20% of fluorescein or fluorescein monoglucoronide that is not bound to plasma proteins. The remaining 80%–90% protein‐bound fraction is expected to be cleared via tubular excretion. The plasma elimination half‐lives of fluorescein are 23.5 min for ‘free’ fluorescein and 264 min for fluorescein glucuronide. Because the fluorescein analogues present themselves in ionised form, they are not reabsorbed by the kidneys and are detectable in the urine for 24–36 h post dose; 90% of elimination occurs within 48 h [20]. Notably, the plasma elimination and renal clearance rates are dependent on the molecular structure and will be different for every molecule.

### Intra‐operative Imaging

After placing the patient in the Trendelenburg position and docking of the da Vinci Xi Surgical System (Intuitive Surgical Inc, Sunnyvale, CA, USA), the surgeon commenced the extended pelvic lymph node dissection, followed by RARP. At three timepoints during RARP, the presence of fluorescein within the surgical field was assessed *in vivo*: (i) immediately before opening the bladder neck; (ii) after opening the bladder neck; and (iii) after removal of the prostate. Because the integrated Firefly endoscope of the da Vinci Xi (white‐light illumination peak (blue) ~460 nm, fluorescence excitation ~800 nm) cannot differentiate between fluorescein and ICG [22], the photodynamic diagnostic settings of a 1 HUB HD + D‐light C light source (Karl Storz, Tuttlingen, Germany) were used to specifically identify fluorescein [23]. In case of contamination with blood or urine, the surgical field was cleared using gauze, suction or irrigation with lukewarm saline (NaCl 0.9%; solubility fluorescein 500 mg/mL) according to standard operating protocol. The presence of fluorescein in the catheter bag was imaged with a normal (white‐light) camera following dye excitation with a standard consumer ultra‐violet flashlight (Conrad Electronic Benelux B.V., Oldenzaal, the Netherlands). The presence of fluorescence was objectively determined as positive or negative without quantification of the signal.

### Statistical Analysis

In this study, descriptive statistics are presented as frequencies with percentages or as medians and interquartile range (IQR), calculated using IBM SPSS version 29.

## Results

Fluorescein could clearly be observed in the urine of the bedside catheter bag after a median (IQR) of 1.31 (1.17–1.72) h (Fig. 1, Table 1). In 2 patients, fluorescein was still observable using an ultra‐violet flashlight in the bedside urine catheter bag 20 h post‐surgery.

### Imaging of Contamination within the Prostatic Bed

**Fig. 2:**
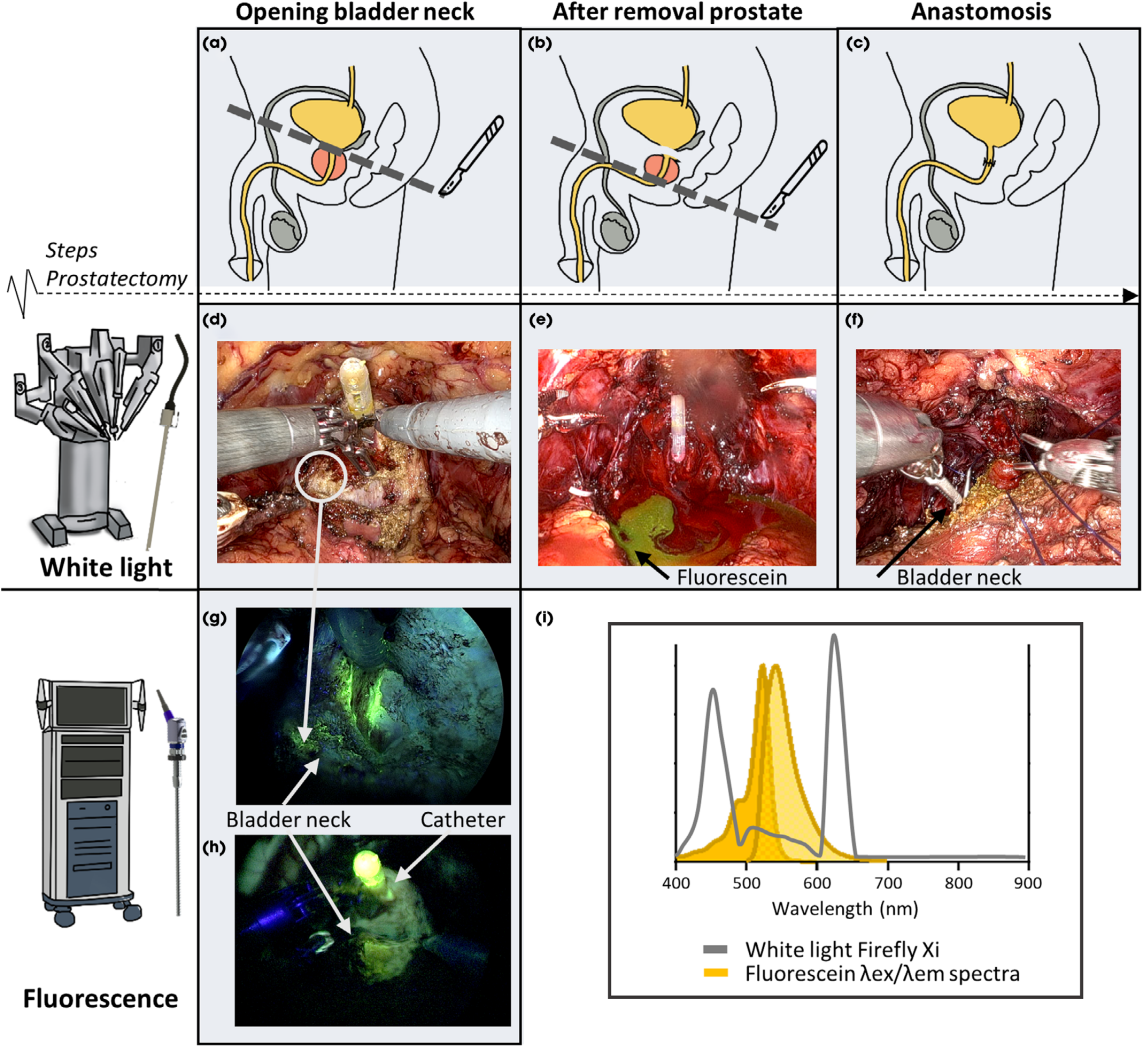
Identification of agent (fluorescein) contamination within the surgical field. (**A**) Schematic lateral view of the male pelvis demonstrating the moment of opening the bladder at the bladder neck during robot‐assisted radical prostatectomy. (**B**) Schematic lateral view of the male pelvis demonstrating the moment of dissecting prostate from the distal urethra. (**C**) Schematic lateral view of the male pelvis demonstrating the anatomy after anastomosis. (**D**) Xi Firefly white‐light imaging of the surgical field after opening the bladder. (**E**) Xi Firefly white‐light image of the prostatic bed after removal of the prostate. (**F**) Xi Firefly white‐light imaging of fluorescein visible (yellow) in the bladder neck during anastomosis. (**G**) Fluorescence imaging using a Karl Storz fluorescence endoscope of the surgical field after opening the bladder. (**H**) Fluorescence imaging using a Karl Storz fluorescence endoscope of the surgical field after opening the bladder showing the catheter tip illuminating. (**I**) The excitation and emission spectra of fluorescein and the white‐light properties of the Xi Firefly system (based on Meershoek et al. [22]).

After opening of the bladder neck, fluorescein could be identified in urine that leaked into the abdominal cavity (Fig. 2E). Although the catheter showed some autofluorescence when imaged with the fluorescence camera, the bright signal observed seemed to be related to residual urine at the catheter tip (Fig. 2H). In 6 patients, fluorescein was visible using the white‐light settings of the Firefly Xi camera. The substantial violet/blue component of the Firefly light source excites fluorescein [22] (Fig. 2E,I). During surgery, fluorescein uptake was clearly observed within the bladder neck tissue in 13/16 patients (Fig. 2G,H). The fluorescent signal in the urine was blocked by the muscular bladder wall until the incision was made. In contrast to this study, in previous studies (which used a 6.25 times higher dose), fluorescein could be observed within the ureters without opening the urinary tract [24].

**Fig. 3:**
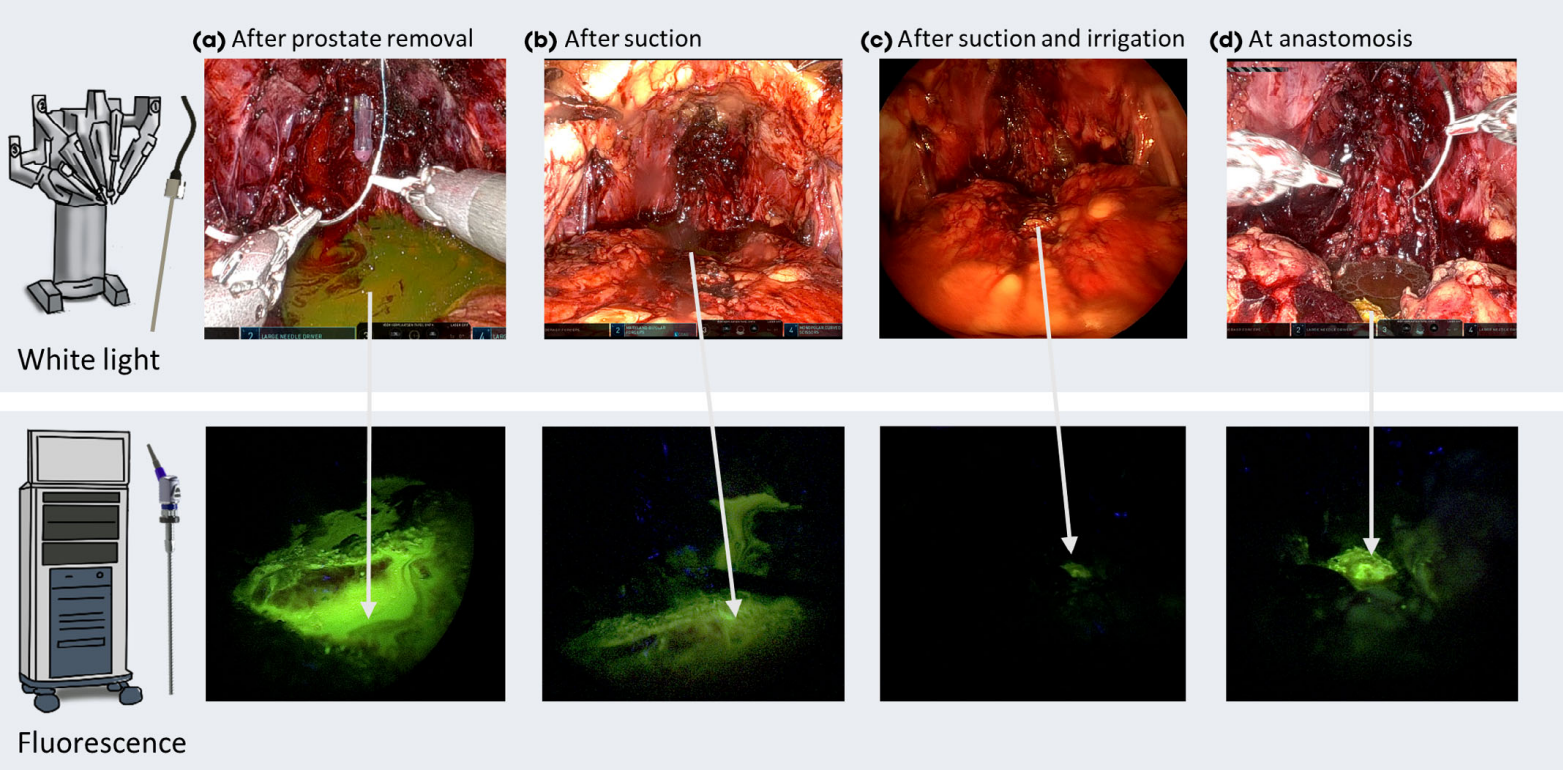
Agent contamination within the surgical field. The agent (fluorescein) contamination is depicted in white light from the Firefly Xi Camera system (top images) and Karl Storz fluorescence endoscope (bottom images). (**A**) Directly after removal of the prostate without cleaning the surgical field, in white light. (**B**) After only suction of the visible contamination. (**C**) After single irrigation with NaCl 0.9% and suction only fluorescein that has been absorbed by the bladder neck remains visible. (**D**) The bladder neck with the absorbed fluorescein remains visible using white light and fluorescence imaging also at anastomosis.

Following the removal of the prostate, the presence of fluorescein could clearly be observed in the prostatic bed in all patients (Fig. 2E). After imaging to show the full extent of the contamination of the surgical field, suction and gauze were used to help to clear the surgical field (Fig. 3). Irrigation of the surgical field with the suction irrigator (if necessary, more than once and, on average, twice) proved most successful for removal of the fluorescein). Despite the high solubility of fluorescein in NaCl 0.9% (up to ~500 mg/mL) [20], it was impossible to wash away all signal (Fig. 3C), with a small fraction remaining at the bladder neck (Fig. 3D). When the bladder was open, new flushes of fluorescein‐containing urine continuously contaminated the view, thus allowing tissue to again absorb the fluorescein. Contamination stopped when the anastomosis was complete (median [IQR] operating duration 244 [222–256.5] min).

## Discussion

By exploiting the slow‐release kinetics of intradermally injected fluorescein it has become possible to study how a renally cleared fluorescent agent can contaminate the surgical field during RARP in a clinical setting. The same may be true for other fluorescent agents known to be cleared via urine, namely, methylene blue, IRDye 800CW and ZW8001 [25]. Monitoring of urine contamination provides insights that are relevant for indications where receptor‐targeted fluorescence imaging is actively being exploited to identify tumour within the urinary tract, for example, extracapsular spread and radical margin resections during RARP [26]. This strategy is only reliable when the observed staining directly relates to the PSMA expression in tumour cells. As fluorescein is not known to bind to tumour receptors, such as PSMA, and was seen throughout the surgical field, our evaluation underscores that caution is required when renally cleared dyes are used to identify margins during fluorescence‐guided RARP. The same caution should apply when receptor‐targeted agents are developed for other cancers of the urinary tract, such as bladder cancer, upper urinary tract cancer, and perhaps even invasive vulva cancer or penile cancer.

The fluorescent staining observed within the bladder neck tissue suggests some diffusion into the bladder wall. This may be explained by the fact that exposure of urine containing (lipophilic) organic molecules promotes dye accumulation within and subsequent staining of the top cell layers of the bladder neck resection area. A well‐known example of this is mitomycin, an agent used to treat bladder carcinoma among other cancers [27]. In fluorescence‐guided surgery, the uptake of agent into the tissue can pose risks; when a surgeon assumes a fluorescent signal is related to the cancerous tissue, non‐tumour‐specific uptake is likely to promote overtreatment. Strikingly, the non‐tumour‐specific accumulation of fluorescein in the bladder neck was consistent with a previous study, in which the non‐tumour‐specific uptake of the renally cleared OTL78 was also observed in the bladder neck [9]. Nguyen et al. [8] also noted non‐tumour‐specific bladder neck uptake, however, in their study they also confirmed tumour presence in a fluorescent bladder neck with a suspicious thickened appearance under white‐light imaging. Studies on Cerenkov luminescence imaging indicate that cauterisation can also influence results, potentially contributing to false positives in the well‐vascularised bladder neck [15]. This effect may occur either because tissue damage from cauterisation allows the agent to penetrate more deeply into the tissue or as a result of chemiluminescence [15, 28].

Fluorescein contamination was partially eradicated using gauze or suction, but irrigation of the prostatic bed had the best effect. Given the prolonged and continuous release of contaminated urine, 48 h in the case of fluorescein, meticulous cleaning of the surgical cavity is required every time a surgeon intends to use fluorescence to identify the tumour extent. Our study findings suggest that the risk of contamination continues despite using a combination of irrigation and suction, and that the signal intensity merely decreases. Regardless of the surgical approach used, the dissection plane always involves the bladder/urethra, meaning the risk of tissue contamination cannot be avoided, although, clearly, contamination of the bladder neck will not be apparent during bladder neck‐sparing procedures. Extending the time between agent administration and surgery beyond the renal excretion window will prevent the presence of contaminated urine during surgery. Various time intervals have been reported in clinical studies describing the use of PSMA‐targeted agents for primary cancer identification; for example, an interval of 17 h for ^99m^Tc‐PSMA I&S [14], 24 h for ^111^In‐PSMA‐I&T [12], OTL78 [9] and IS‐002 [8], and 72 h for IR800‐IAB2M [11]. That said, the biological half‐life (*t*_1/2 biol_), a feature unique to every molecular design, is a time frame within which urine containing imaging agent has not been reported for any of the PSMA agents. Another key parameter to take into consideration with regard to the biological half‐life is that the absolute amount of detectable agent in the urine is highly dependent on the injected dose. When micro‐dosing of ^99m^Tc‐PSMA I&S and ^111^In‐PSMA‐I&T is used (<100 μg/patient), the absolute signal will rapidly drop below detectable levels. The therapeutic dosing used with OTL78 (30 μg /kg [9]), IS‐002 (25 μg/kg [8]) and IR800‐IAB2M (50 μg/kg [11]) means that substantially more time is needed to allow the signals to drop below detectable levels, a feature that has already motivated the use of dose‐reduction studies for these agents to diminish background signals [8, 9, 11]. Dose reduction, however, may in some cases also impair the identification of the targeted tissue [8].

A conceptual limitation of this study is that we were trying to understand and mitigate the risks of using renally cleared agents to identify lesions within the urinary tract, instead of focusing on designing hepatically cleared agents that would circumvent such issues [5]. This is especially notable given that the value of hepatic clearance of PSMA agents has already been demonstrated; ^18^F‐PSMA‐1007 is said to increase the diagnostic confidence offered by PET/CT when interpreting lesions adjacent to the urinary tract, for example, local recurrence [29, 30]. This shift from renal to hepatic clearance has also been proposed for intra‐operative PSMA‐targeted agents [31], a molecular design that appears to warrant further exploration. Limitations of this study include that 80 mg (1.14 mg/kg) intradermal injections of fluorescein, a dye with a relative short biological half‐life (*t*_1/2 biol_ = 23.5 min after i.v. injection), can only provide an indication of what urine contamination with receptor‐targeted agents may entail. This approach may lead to over‐ or underestimation of the fluorescent urine content resulting from i.v. administrated doses (see doses of known agents above) at the time of surgery. As fluorescein is relatively inert and easy to rinse away with water, the irrigation results may not imply the success of this approach with other agents. Conversely, tissue attenuation may limit the identification of fluorescein at 1 mm below the tissue surface, while agents functionalised with other dyes may be detected in tissue up to 1 cm deep, thus yielding potentially stronger non‐tumour‐specific signals [32]. It is unclear how local exposure of the bladder neck to imaging agent‐containing urine can lead to absorbance of fluorescein. Furthermore, in this relatively small patient cohort, interpatient variability in metabolism and renal clearance rates of fluorescein may have influenced the average time between injection and the visibility of fluorescein within the surgical field. As we used fluorescein merely as a model system of urinary clearance, we recommend that future studies on PSMA agents investigate the urinary clearance and contamination in more detail.

In conclusion, by monitoring fluorescein content within urine during RARP, this study showed that renally cleared agents contaminate the surgical field as soon as the bladder neck is opened up until the anastomosis is complete. This yielded non‐tumour‐specific uptake in the bladder neck; it should therefore it should be kept in mind that renally cleared agents could compromise the accuracy of intra‐operative imaging during RARP. Although irrigation and suction helped to substantially reduce the fluorescein contamination, other cleaning methods may be required for other fluorescent agents.

In summary, renally cleared agents may interfere with fluorescence‐guided tumour margin assessment in the urinary tract.

## Disclosure of Interests

F.v.L. is a recipient of a Dutch Society of Science grant that helped made this work possible. This grant was co‐sponsored by Intuitive and Karl Storz. Karl Storz and Intuitive had no influence on the scientific output. H.v.d.P. has been supported by Intuitive, Janssen, Ipsen, Astra Zeneca, Karl Storz, Accord Healthcare, and On‐target Laboratories, none of which played a direct role in this study.
